# Positive Influences of Ohmicsonication on Phytochemical Profile and Storage Stability of Not-from-Concentrate Mango Juice

**DOI:** 10.3390/molecules27061986

**Published:** 2022-03-18

**Authors:** Tarek Gamal Abedelmaksoud, Sobhy Mohamed Mohsen, Lene Duedahl-Olesen, Ammar B. Altemimi, Mohamed Mohamed Elnikeety, Francesco Cacciola, Aberham Hailu Feyissa

**Affiliations:** 1 Food Science Department, Faculty of Agriculture, Cairo University, Giza 12613, Egypt; sobmohsen1@hotmail.com (S.M.M.); mnoketi@yahoo.com (M.M.E.); 2 National Food Institute, Technical University of Denmark, 2800 Kgs. Lyngby, Denmark; lduo@food.dtu.dk (L.D.-O.); abhfe@food.dtu.dk (A.H.F.); 3 Food Science Department, College of Agriculture, University of Basrah, Basrah 61004, Iraq; ammar.ramddan@uobasrah.edu.iq; 4 Department of Biomedical, Dental, Morphological and Functional Imaging Sciences, University of Messina, Via Consolare Valeria, 98125 Messina, Italy

**Keywords:** ohmicsonication, mango juice, microbial load, polyphenoloxidase, carotenoids

## Abstract

Processing technique and storage conditions are the main factors that affect the phytochemical profile of Not-from-Concentrate (NFC) juice, which could decrease the nutritional and bioactive properties of the corresponding juice. The aim of this study was to evaluate the quality changes that occurred in NFC mango juice after Ohmicsonication (OS) and during storage in comparison to other processing methods such as sonication (S), thermosonication (TS), ohmic heating (OH), and conventional heating (CH). Quality attributes such as polyphenoloxidase (PPO) and pectinmethylesterase (PME) activities, ascorbic acid and hydroxymethyl furfural (HMF) contents, total phenolics, total flavonoids, total carotenoids, electric conductivity, color values and microbial load (total plate count, mold, yeast, and psychrophilic bacteria) were examined. OS and OH treatments demonstrated the highest inactivation of PPO (100%), while CH and TS displaying inhibitions 89% and 90%, respectively and only S treatment exhibited insufficient inactivation of both PPO and microbial load. However, the inhibition of PME followed the order OS (96.5%) > OH (94.9%) > TS (92.5%) > CH (88.5%). The best treatment, with the highest retention of phytochemical contents (ascorbic acid, total carotenoids, antioxidant activity, total flavonoids, and total phenolic content) for NFC mango juice and during storage was obtained with OS treated samples compared to other treatments (in the order from the lowest to highest percentage, OS < OH < TS < CH). Consequently, the results indicated that OS could be applied as a new mild thermal treatment in the production of mango juice with improved quality properties of stored NFC mango juice.

## 1. Introduction

According to several studies, different fruit juices show a distinct rate of quality degradation during storage [1,2]. Consequently, the fruit juice composition plays an important role in its stability. However, even after processing with the aim of shelf-life extension, it is known that food product quality is not constant, or in other words, it changes continuously over time. Even for a shelf-stable product, there is a limitation in its shelf life due to deteriorative chemical reactions determining its best-before date [3,4]. Juice processed from Not-from-Concentrate (NFC) is one of the preferable juices in the market. The term NFC is used for the natural juice after removal of the foreign impurities (i.e., insoluble pulp, skin, and seeds) and thermal treatment for the reduction of both microbial load and enzymatic activities which reduce storage quality [5].

Mango (*Mangifera indica* L.) is a tropical fruit with a distinctive flavor, color, and high content of nutrients. Mango has a rich composition due to its content of carotenoids, phenolic compounds, ascorbic acid, sugar and minerals, fibers, and other organic compounds [6]. Mango has been reported to constitute health effects by consumption to avoid chronic diseases linked to oxidative stress, such as cardiovascular disease, cancer, and neurodegenerative diseases [7,8]. It is cultivated in Asia, Africa, and America in many tropical and subtropical countries [9]. Mango fruit is a perishable commodity, which should be processed into other products to prolong its shelf life. To extend the shelf life of mango products and for storage at ambient temperature for a long time, the conventional thermal treatment can be used. Many thermally treated mango products are available in the market such as mango juice, puree, jam, canned slices, and nectar [10]. In this paper, we will focus on mango juice processing to inactivate the enzymatic and microbial activities in the juice in order to extend its shelf life. These activities must be in control during juice processing steps [11], to prevent undesirable effects e.g., browning by polyphenoloxidase (PPO) as well as phase separation caused by pectic enzymes especially pectinmethylesterase (PME).

At the same time, fruit juices are thermally sensitive and susceptible to chemical, physical, and microbiological changes during harvesting, processing, and storage. Preservation of fruit juice by high-temperature short time (HTST) treatment is commonly used to reduce microbial load, unfortunately with concomitant quality reduction by color alterations, cloud formation, flavor damages, vitamins, and other nutritional losses at especially temperatures above 80 °C [12,13]. Consumer’s request for fruit juices with high nutritional value and quality standards that are closer to fresh without additives and preservatives has stressed the use of hurdle techniques based on reduced temperatures.

Ohmicsonication (OS) is defined as a combination of sonication with OH as a substitute pasteurization process for obtaining the quality properties of juices. The effect of OS treatment on overall quality characteristics of apple and orange juices was evaluated by the authors. These studies reported that OS led to more retention of bioactive components as well as more inactivation for PPO and PME activities than conventional heating techniques [14,15]. Therefore, during this study, we focus on the effects of OS on the quality characteristics and storage stability of NFC mango juice. After optimization of OS by RSM as a potential quality enhancement technique of NFC mango juice, the quality characteristics were studied. We also investigated the effect of OS on physical, chemical, and microbiological characteristics of NFC mango juice during cold storage for 8 months (under modified atmosphere conditions).

## 2. Results and Discussion

### 2.1. Optimization of Ohmicsonication (OS) Parameters to Inactivate PPO and PME in NFC Mango Juice

Working parameters of OS application were determined by pretesting. Second-order polynomial models were used for response (PPO and PME activity) to determine the specified optimum conditions. The obtained optimum parameters were 75 °C for 8 min in mango juice for 100% PPO inactivation), and a PME inactivation of 98% in NFC mango juice (data in supplementary material).

### 2.2. Phytochemical Profiles, Enzymatic Activities, and Microbial Load of Fresh and Processed NFC Mango Juice

The effects of different treatments (i.e., S, CH, TS, OH, and OS) on phytochemical contents (i.e., ascorbic acid content, total phenolic contents (TP), total flavonoids content (TF), total carotenoids content (TCC), antioxidant acidity and hydroxymethylfurfural (HMF) and enzymatic activities (i.e., PME and PPO), as well as microbial load of NFC mango juice, are presented in Table 1.

The results show a significant (*p* ≤ 0.05) decrease in the ascorbic acid content of NFC mango juice in all the treatments (S, CH, TS, OH, OS) compared to fresh mango juice (FMJ). The highest ascorbic acid content was obtained with S followed by OS, OH, TS and CH. The decrease in the ascorbic acid content of NFC mango juice in the treated samples (OS, OH, TS and CH) was due to the effect of heat and processing time that might induce chemical decomposition of ascorbic acid at higher rates. These results are similar to Demirdöven and Baysal [16], who observed a decrease of ascorbic acid in the OH and CH of orange juice compared with control group.

A significant (*p* ≤ 0.05) increase in the total carotenoids of sonicated NFC mango juice (2.3%) was probably due to the mechanical disruption of cell walls, which might enhance free carotenoids in the juice as suggested for orange juice [17]. This agreed with Abid et al. [18] who observed an increase in the total carotenoids with sonication of apple juice compared to non-sonicated juice samples. A significant decrease of carotenoids was observed for OS, TS, OH, and CH treatments, which might be due to extensive losses of carotenoids due to heat treatment used in each processing technique [10,19].

The total phenolic (TP) content and phenolic compounds (gallic acid, chlorogenic acid, caffeic acid, rutin, *p*-coumaric, ferulic acid, quercetin, and naringenin) of NFC mango juice was significantly increased in all treatments compared to fresh mango juice (FMJ): from the highest to the lowest TP content in the order of S > OS > TS > OH > CH. The increase of TP during heating could be attributed to increased extractability of total phenolic components due to the changes in the tissue matrix induced by high temperatures [20] and disruption of complexes between polyphenols and proteins [21]. During ohmic heating, the alternating current has a synergistic effect on releasing total phenolic contents resulting in a slight increase for phenolic content previously illustrated for broccoli [22]. Also, ultrasonic-treated orange extracts were characterized by significantly (*p* < 0.05) increased TP values with increased ultrasonic time, power, and temperature [23].

The antioxidant activity of juice is commonly attributed to the contents of phenolics, flavonoids, ascorbic acid, and carotenoids. Excluding S treatment (68.7%), the antioxidant activity (%) of NFC mango juice was significantly decreased in all treatments compared to fresh mango juice (FMJ): from the highest to the lowest antioxidant activity % in the order of OS > TS > OH > CH. This agreed with the values of phenolics, flavonoids, ascorbic acid, and carotenoids (Table 1). Ohmicsonication (OS) treated NFC mango juice contained the lowest value of HMF (6.90 mg/L) followed by OH (7.27 mg/L) and TS (8.32 mg/L) and CH (8.71 mg/L) treated juice contained the highest value of HMF. These levels for HMF were within the expected range (not detected to 27.3 mg/L) reported for fruit juice in general [24].

The presented data (Table 1) shows a significant decrease in the PPO and PME activity in both S, CH, TS, OH, and OS compared to FMJ. The PPO activity was completely inactivated with OH and OS treatments, while the 38%, 89%, and 90% inhibition were seen for S, CH, and TS, respectively. The complete inactivation by OH and OS treatments might be due to the nature of PPO enzyme in mango juice, which results in a low heat stability (isozyme with a lower Thermostability) [25]. The inactivation of PPO in mango juice significantly increase at about 50–60 °C according to [26] and this temperature is more less compared to other fruits like apple, pear, avocado, and plum (60–65 °C) [27]. The inhibition of PPO by the TS and CH was probably due to the thermal effect that caused denaturation of the enzyme and for TS additional effects of cavitation during the sonication were obtained. The increase of the PPO inhibition was probably due to an electric field that might remove the metallic prosthetic groups present in the PPO, resulting in the enhancement of enzyme activity loss [28]. Therefore, the inhibition of PPO in the OS was due to the combined effect of cavitation during sonication, thermal effects during heating, and electric field application during OH treatment [29].

PME activity was significantly reduced in all treatments (S, OH, TS, OH, and OS) when compared to the fresh sample (FMJ in Table 1). The highest reduction (inhibition) in the PME activity was obtained by OS treatment (96.5%) followed by OH (94.9%), TS (92.5%), CH (88.5%), and S (71%). These results confirm that PME was more resistant to heat than PPO and previously thermal inactivation of PME in mango was found to range from 64 to 78 °C [30]. The present results confirmed that sonication alone was not sufficient to reduce the PPO and PME activity in the mango juice. In the literature, no study was found on OS or OH of mango juice, however, two studies on oranges were based on OH [16,31]. Both studies found results similar to the present study, namely a larger reduction (compared to fresh orange juice) in the PME activity during ohmic heating compared to CH. In general, for both enzymes (PPO and PME) the reduction of activity was due to the effect of heating during OH and CH in addition to the effect of application of an electric field for OH, which could influence biochemical reactions by changing the molecular spacing and increasing interchain reactions due to the OH treatment [29].

Results on microbial load (TPC, PB, and mold and yeast) showed no detected growth for all treatments except for fresh mango juice (2.5 log cfu/mL) and S (1.6 log cfu/mL). In comparison, Mohsen et al. [32] found that molds and yeast as well as TPC counts of fresh mango juice were 4.51 log cfu/g and 5.76 log cfu/g, respectively. They also found that after processing of mango pulp by OH the TPC, as well as mold and yeast growth, were completely inhibited, while for CH, TPC had only 3.14 log cfu/g and 2.07 log cfu/g activity in mango pulp. After 12 months of storage, they found that OH samples had no M and Y activity in mango pulp, while in the CH samples activity was 2.57 log cfu/g. On the other hand, TPC values after 12 months of storage were 1.07 log cfu/g and 4.06 log cfu/g in OH and CH of mango pulp samples, respectively [32].

### 2.3. Phytochemical Profiles and Enzymatic Activities of Stored NFC Mango Juice

**Figure 1:**
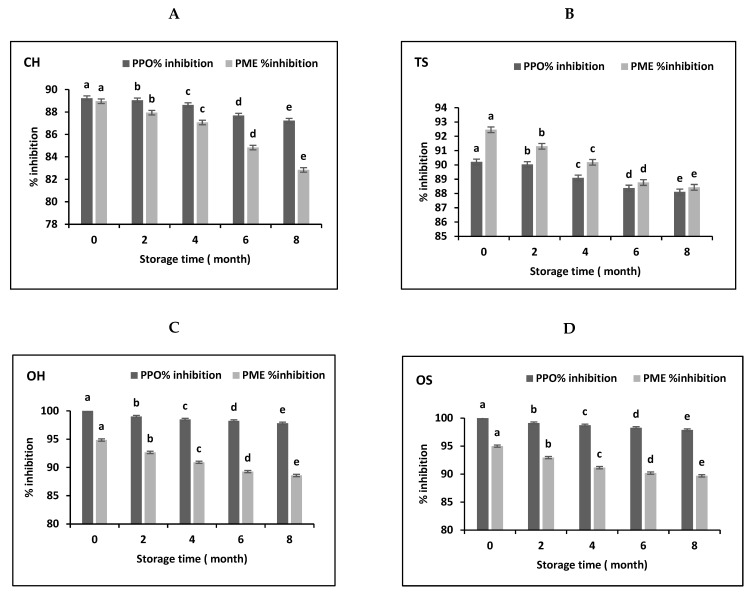
Effect of storage time (0, 2, 4, 6 and 8 months) on PPO and PME activity in percent of NFC mango juice treated by CH (**A**), TS (**B**), OH (**C**) and OS (**D**); a, b, c, d, e indicate statistical significance compared with the control, as determined by Student’s *t*-test (*p* < 0.05); data are means ± S.D. (*n* = 3). PPO: polyphenoloxidase; PME: Pectinmethylesterase; OS: Ohmicsonication; TS: Thermosonication; OH: ohmic heating; CH: conventional heating.

The effect of storage of CH, TS, OH and OS treated mango juice at 2 ± 2 °C under modified atmosphere conditions for 8 months with sampling and analysis at two months’ intervals for CH, TS, OH, and OS treatments are presented. In Figure 1 data on PME and PPO activities are reported. Results indicate a significant decrease (*p* ≤ 0.05) in the % inhibition of PME and PPO of stored CH, TS, OH and OS treated juices. The % decrease of PME activity in NFC mango juice from 89%, 92%, 95% and 95% to 83%, 88%, 89% and 90%, respectively. On the other hand, the % decrease in PPO inhibition in NFC mango juice from 89%, 90%, 100% and 100% to 87%, 88%, 98% and 98%, respectively.

**Figure 2:**
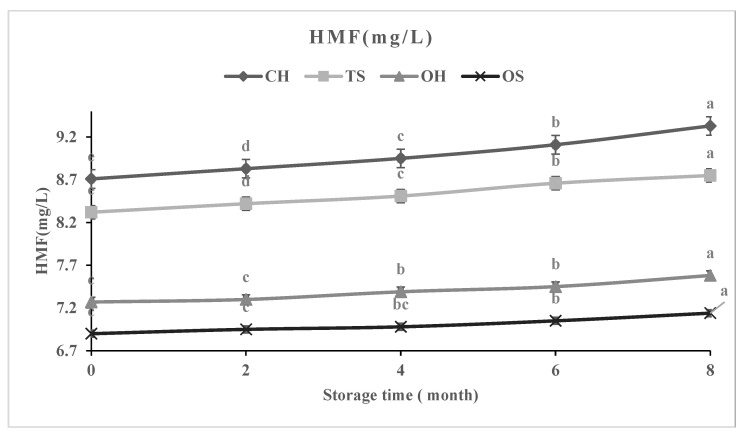
Effect of storage time (0, 2, 4, 6, 8 months) on HMF concentration of NFC mango juice. HMF: Hydroxymethyl furfural; OS: Ohmicsonication; TS: Thermosonication; OH: ohmic heating; CH: conventional heating. Within lines, different letters indicate statistical differences (*p* < 0.05); data are means ± S.D. (*n* = 3).

**Figure 3:**
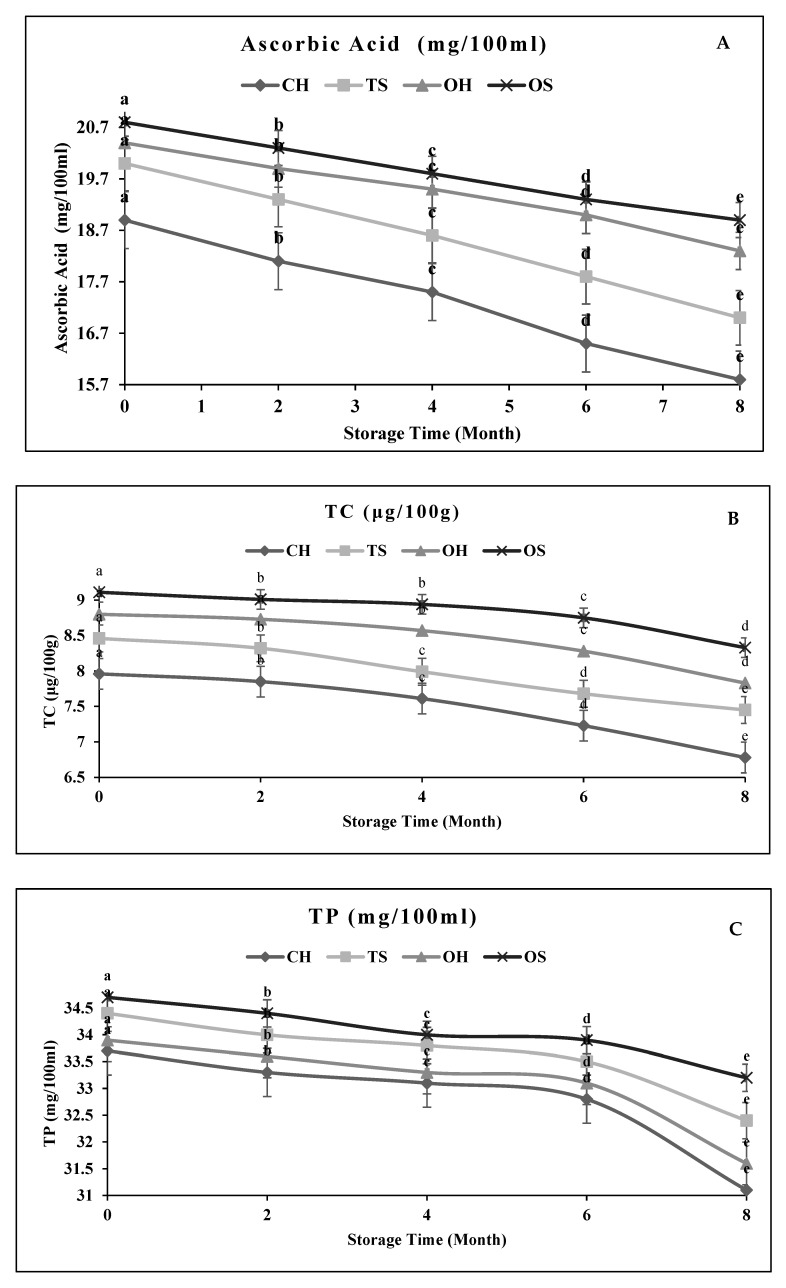

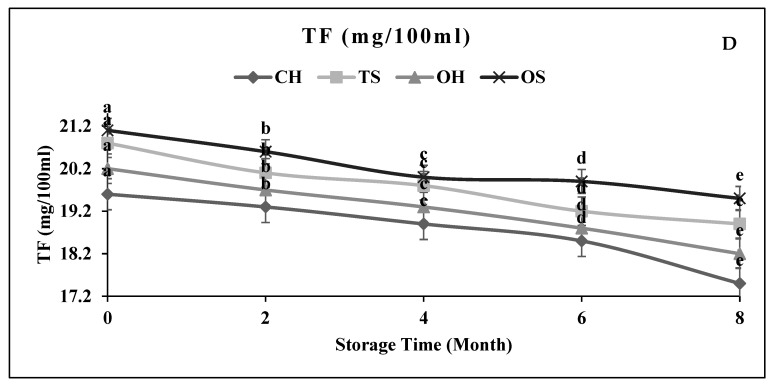
Effect of storage time (0, 2, 4, 6, 8 months) on ascorbic acid (**A**), TC (**B**), TP (**C**) and TF (**D**) contents of NFC mango juice. TP: total phenolic; TF: total flavonoids content; TCC: total carotenoids content; OS: Ohmicsonication; TS: Thermosonication; OH: ohmic heating; CH: conventional heating. Within lines, different letters indicate statistical differences (*p* < 0.05); data are means ± S.D. (*n* = 3).

During storage, the highest increase (%) in HMF values (Figure 2) was detected in NFC mango juice treated with CH (7.10%) followed by TS (5.16%), OH (4.26%), and OS (3.47%). The phytochemical profile during storage for 8 months with analysis every 2 months in Figure 3 show a slight decrease in ascorbic acid, total carotenoids (TCC), antioxidant activity, total flavonoids (TF), total phenolic content (TP), and phenolic acids of NFC mango juice processed by TS, CH, OH, and OS. The best treatment, with the highest retention of phytochemical contents (ascorbic acid, total carotenoids, antioxidant activity, total flavonoids, and total phenolic content) for NFC mango juice was obtained with OS treated samples compared to other treatments (in the order from the lowest to highest percentage, OS < OH < TS < CH).

The decrease in ascorbic acid contents due to storage at 2 ± 2 °C for 8 months of NFC mango juice was 9.13% by OS; 10.29% by OH; 15.00% by TS and 16.40% by CH, respectively. The reduction % in total carotenoids values of NFC mango juice was 8.56% by OS; 11.02% by OH; 11.94% by TS and 14.82% by CH, respectively. The decrease in TP and TF (%) of NFC mango juice was 4.32% and 7.58% by OS; 5.81% and 9.13% by OH; 6.78% and 9.90% by TS 7.72% and 10.71% by CH, respectively. In this regard, Alaka et al. [33] also reported a decrease in ascorbic acid contents of mango juice due to storage at 6 °C for 8 weeks. Also, Van den Berg et al. [34] observed that mango pulp treatment by conventional heating caused degradation in colored pigments (β- carotene).

## 3. Materials and Methods

### 3.1. Chemicals

Citrus pectin, Catechol, polyvinyl poly pyrrolidone (PVPP), 2,6-dichlorophenol-indophenol (DCPIP), sodium bicarbonate, L-ascorbic acid, fructose, sucrose, glucose, 5-hydroxy methyl furfural, Aluminum Chloride, Sodium hydroxide, Sodium nitrate, Butylated hydroxytoluene (BHT), Oxalic acid, Folin-Ciocalteau reagent and sodium carbonate from (Sigma-Aldrich Chemical Co., Brøndby, Denmark), Hexane, Acetone, Methanol from Sigma and Fluka and NaOH 50% (*w*/*w*) (J.T. Baker 7067, NJ, USA) were used.

### 3.2. Raw Material

**Figure 4:**
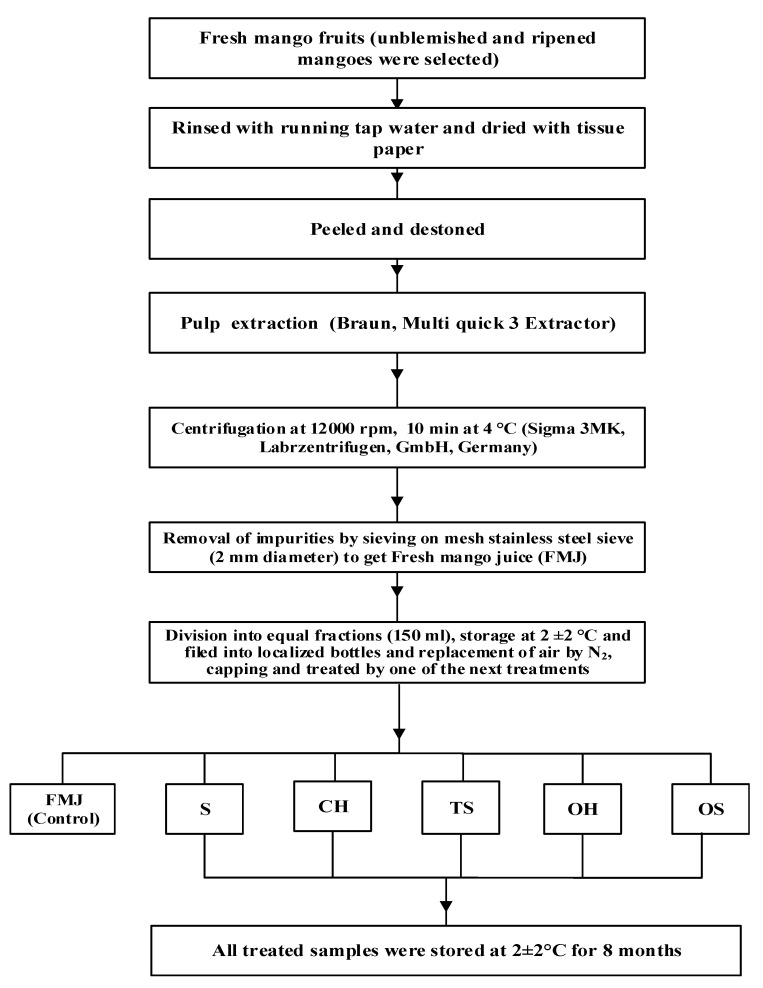
NFC mango juice processing diagram.

Fresh mango (*Mangifera indicia* L., cultivar Kent) fruits were purchased from a local supermarket in Copenhagen, Denmark. They were stored in a refrigerator at 7 °C at 80–90% humidity for a maximum of 48 h before processing. The flowsheet (Figure 4) illustrates the experimental procedure of the mango juice processing with different methods.

### 3.3. Processing Methods

#### 3.3.1. Conventional Heating (CH)

Samples of mango juice (150 mL) were heated at 90 °C for 60 s in a clean 250 mL glass bottle using a shaker water bath (Julabo SW22, Julabo GmbH, Seelbach, Germany), according to Abedelmaksoud et al. [35].

#### 3.3.2. Sonication (S) and Thermosonication (TS)

Sonication was performed on 150 mL mango juice in a 250 mL glass bottle. The ultrasonic processor of 550 W (Branson SFX550 Sonifier, Nuevo Laredo, Mexico), with a 0.5-inch probe operating at 20 kHz frequency was used for sonication. Samples were treated at 60 °C for thermosensation and less than 25 °C for sonication by radiating 100% of power (550 W) for 8 min and, keeping pulse durations of 5 s. Overheating of the samples was prevented by circulating ice water through the treatment chamber. Thermosonication (TS) of samples was started when the set temperature at 60 °C. The observed rise in temperature of the samples due to sonication was 2–5 °C.

#### 3.3.3. Ohmic Heating

The treatment of NFC mango juice (150 mL) by ohmic heating (at 40 V/cm until 80 °C and held for the 60 s) was conducted according to Abedelmaksoud et al. [5].

#### 3.3.4. Ohmicsonication

Ohmicsonication (OS) was conducted by a combination of ohmic heating and sonication treatments, where the mango juice samples were first treated by radiating 100% of power (550 W) for 8 min by sonication directly followed by ohmic heating at 40 V/cm until 75 °C for the 60 s.

#### 3.3.5. Experimental Design

The influence of OS parameters (sonication time (min) and OH temperature (°C)) on the activity of PPO and PME by response surface methodology (RSM) was investigated. For two factors (OH temperature and sonication time) with three levels (−1, 0, +1), the factorial design (3^2^) was used. The OH temperature and sonication time range were 55, 65 and 75 °C and 2, 5, 8 min, respectively (Table S1). To describe the effect of OH temperature and sonication time, the second-order polynomial model was used (Equation (1)).

(1) $$Y=a_{o}+a_{1}x_{1}+a_{2}x_{2}+a_{12}x_{1}x_{2\;\;}+a_{11}x_{1}^{2}+a_{22}1x_{2}^{2}$$

where, *Y* is the PME activity, *x*_1_ is OH temperature and *x*_2_ is sonication time, *a_o_*, *a*_1_, *a*_2_, *a*_11_, *a*_22_ and *a*_12_ are regression coefficients for intercept, the linear, the quadratic and interaction term, respectively. The analysis of variance (ANOVA) for the response (activity of PME) was used to find the significant terms in the models (Table S2). Design Expert Version 10.0.6 software was used for the analysis. To optimize the OS parameters, the desirability function method according to Derringer & Suich [36] was used. The objective function is to maximize the PME inactivation using desirability function as described by Abedelmaksoud et al. [35]. Each treatment was repeated three times; means and standard deviations of results were calculated.

#### 3.3.6. Phytochemical and Enzymatic Analysis

The ascorbic acid content and total phenolic content were determined as the protocol described by Abedelmaksoud et al. [37]. The obtained results were expressed as mg of ascorbic acid per 100 mL sample and total phenolic contents of the samples were expressed as mg of Gallic acid per 100 mL.

Total carotenoids content was measured at 450 nm according to hexane/methanol/acetone extraction by Lee and Castle [38] with modifications as described in Abedelmaksoud et al. [35] for apple juice. The total carotenoid contents were calculated using a β-carotene extinction coefficient of, E1% = 2505 (µg β-carotene/100 g) according to Ritter & Purcell (1981) as previously stated.

Total flavonoids content was measured by a colorimetric assay developed by Kim et al. [39], with modifications. Briefly, 250 µL of extract or standard solution of catechin at different concentrations (20–260 µg/mL) and 1 mL of distilled water were mixed in a 10 mL test tube. The following were successively added: at time zero, 75 µL of 5% NaNO_2_; at 5 min, 75 µL of 10% AlCl_3_; and at 6 min, 500 µL of 1 N NaOH. The solution was then immediately diluted by adding 2.5 mL of distilled water and mixed thoroughly. The absorbance of the mixture (pink color) was directly measured by a microplate reader at 510 nm against a blank sample and the results were expressed as catechin equivalents (mg CE/g).

Hydroxymethyl furfural (HMF) was determined according to Kalábová and Večerek [24]. A half ml of juice was mixed with 1 mL methanol on a Vortex Genie II (Scientific Industries INC, Bohemia, NY, USA) for 10 min and centrifuged at 13000 rpm for 10 min. This extract was filtered (0.45 µm) and directly used for HPLC analysis. The chromatographic determination was carried out on an Alliance apparatus manufactured by Waters Company, with a 2996 diode array detector. Zorbax Eclipse XDB-C8, 4.6 × 150 mm, 5 μm column was used (Waters, Milford, MA, USA). The mobile phase (10% methanol in water) flow rate was 1 mL min^−1^, with the sample injection volume of 20 μL and the column temperature of 30 °C. HMF was detected in the UV region at 285 nm. The external standard method was used for the determination of the HMF content using the Empower software (Waters). The retention time for HMF was 3.17 min and the linearity of the HPLC method used was tested in the concentration range of 0.01–200 mg L^−1^ using an HMF standard.

Pectin methyl Esterase activity (PME), Quantitative measurement of PME was based on the methods reported by Rouse and Atkins [40] and Ting and Rouseff [41]. Juice samples were mixed by inverting the bottle several times and 2 mL was transferred into 20 mL of a 1% citrus pectin substrate solution in 0.2 M sodium chloride. The sample was titrated to pH 7.5 with 0.2 N NaOH. An auto-titrator (Dos Bio-5, 665 Dosimat, made by metrohm, Swiss) was used to deliver 0.05 N NaOH to the sample to maintain the pH at 7.5 for 10 min during hydrolysis at 30 °C. The volume of 0.05 N NaOH consumed during this time was recorded. The PME activity expressed as PME units (PMEu) per gram was calculated by using the formula:

PME(U/g/min) = ((mL of NaOH) × (normality OH)/((weight of sample) × (10 min)) × 10^4^

Polyphenoloxidase activity (PPO) was determined by the method of Elsayed et al. [42] with a modification., 5 mL mango juice was mixed with 5 mL of 0.2 M sodium phosphate buffer (pH 6.8) containing 2% (*w*/*v*) polyvinyl poly pyrrolidone (PVPP) and then centrifuged (Sigma 3MK Labrzentrifugen GmbH, Osterode am Harz, Germany) at 10,000 g, 4 °C for 30 min. The supernatant was collected for the enzyme assay. The standard reaction mixture contained 2 mL of 0.05 M catechol in 0.05 M sodium phosphate buffer (pH 6.8), and 1 mL of extract, followed by incubation for 3 min at 25 °C. The increase in absorbance at 420 nm (Microplate reader) was measured and compared with a control cell in which the enzyme extract was substituted by water. PPO activity (1 unit) was defined as an increase in the absorbance of 0.001 min^−1^.

#### 3.3.7. Microbiological Load

Total plate count and psychrotrophic bacteria were determined using the pour plate method; plate count agar was used as a medium and plates were incubated at 35 °C for 48 ± 2 h for total plate count and in the refrigerator at 5 ± 2 °C for 10 days for psychrotrophic plate count [43,44]. Mold and yeast were determined using potato dextrose agar medium, plates were incubated in the dark at 22–25 °C for 5 days [44].

#### 3.3.8. Statistical Analysis

Phytochemical, enzymatic activities, and microbial load results were statistically analyzed by one-way analysis of variance (ANOVA) using the software SPSS 13 (SPSS Inc., Chicago, IL, USA) with the Duncan test to evaluate differences between treatments at levels of significance (*p* ≤ 0.05). Each analysis was repeated three times; means and standard deviations of results were calculated.

## 4. Conclusions

Based on the results of this study, Ohmicsonication as novel combined technologies resulted in the highest inactivation of PPO and PME activities and microbial load with lower losses of phytochemical profiles (i.e., ascorbic acid, carotenoids, phenolic, and flavonoids) compared to other treatments (i.e., ohmic heating, Thermosonication, conventional heating and sonication). Sonication treatment alone was shown to be insufficient for the inactivation of both PPO and microbial load. The results also indicated that Ohmicsonication was the best treatment for the storage period of up to 8 months studied, due to more retention of the phytochemical profiles. Therefore, quality characteristics for Ohmicsonication were more acceptable compared to other treatments, by improving juice quality and shelf life, highlighting the novelty of the present study. It is concluded that Ohmicsonication can be employed as a preservation technique for processing mango juice on a laboratory scale and the possibility of application in a bigger pilot plant could be interesting.

## Figures and Tables

**Table 1:** Phytochemicals profiles, Enzymatic activities and microbial load of fresh mango juice (FMJ) treated by S, CH, TS, OH, and OS.

Parameters	FMJ	S	CH	TS	OH	OS
Ascorbic acid (mg/100 mL)	23.39 ± 0.74 ^a^	21.05 ± 0.08 ^b^	18.93 ± 0.15 ^e^	19.96 ± 0.12 ^d^	20.37 ± 0.23 ^c^	20.75 ± 0.14 ^c^
TCC (μg/100 g)	9688.29 ± 37.72 ^b^	9912.95 ± 53.14 ^a^	7963.12 ± 39.53 ^f^	8661.84 ± 46.56 ^e^	8808.82 ± 24.48 ^d^	9119.55 ± 54.98 ^c^
Antioxidant activity (%)	65.57 ± 2.02 ^e^	68.74 ± 3.40 ^a^	59.97 ± 0.96 ^f^	63.74 ± 3.66 ^c^	61.62 ± 1.99 ^d^	64.37 ± 3.28 ^b^
TF (mg/100 mL)	18.93 ± 0.18 ^f^	21.81 ± 0.32 ^a^	19.60 ± 0.15 ^e^	20.83 ± 0.31 ^c^	19.96 ± 0.24 ^d^	21.14 ± 0.27 ^b^
TP (mg/100 mL)	32.36 ± 0.13 ^f^	35.97 ± 0.31 ^a^	33.48 ± 0.05 ^e^	34.06 ± 0.23 ^c^	33.63 ± 0.17 ^d^	34.358 ± 0.226 ^b^
HMF (mg/L)	nd	nd	8.71 ± 0.03 ^a^	8.32 ± 0.05 ^a^	7.27 ± 0.06 ^b^	6.90 ± 0.04 ^c^
PPO (U/mL/min)	44.65 ± 2.66 ^a^	27.87 ± 2.42 ^b^	4.81 ± 0.55 ^c^	4.37 ± 0.87 ^c^	nd	nd
PME (U/mL/min)	20.57 ± 0.61 ^a^	14.76 ± 0.89 ^b^	2.27 ± 0.20 ^c^	1.55 ± 0.07 ^d^	1.06 ± 0.05 ^e^	0.73 ± 0.05 ^f^
TPC (log cfu/mL)	2.55 ± 0.02	2.23 ± 0.07	nd	nd	nd	nd
PB (log cfu/mL)	nd	nd	nd	nd	nd	nd
M and Y (log cfu/mL)	2.48 ± 0.02	1.58 ± 0.22	nd	nd	nd	nd

^a, b, c, d, e, f^ indicate statistical significance compared with the control, as determined by Student’s *t*-test (*p* < 0.05); data are means ± S.D. (*n* = 3); nd: not detected; TP: total phenolic; TF: total flavonoids content; TCC: total carotenoids content; HMF: hydroxyl methyl furfural; PPO: polyphenoloxidase; PME: pectinmethylesterase; TPC: total plate count; PB: psychrophilic bacteria and M and Y: mold and yeast, FMJ: Fresh mango juice; S: Sonication; CH: conventional heating; TS: Thermosonication; OH: ohmic heating; OS: Ohmicsonication.

## Data Availability

Data is contained within the article.

## Supplementary Materials

The following supporting information can be downloaded at: https://www.mdpi.com/article/10.3390/molecules27061986/s1, Table S1: Ohmicsonication (high power) and PPO and PME activity in mango juice, Table S2: ANOVA for Response Surface Quadratic model (PPO and PME of mango juice), Figure S1: Effect of Ohmicsonication (OS) parameters (temperatures and time) on the PPO and PME activity of mango juice (U/mL/min)–response surface and contour plots. Blue indicates lower PPO and PME activity and red indicates higher PPO and PME activity.

## Abbreviations

FMJ	Fresh mango juice;
NFC	Not-from-Concentrate;
OS	Ohmicsonication;
S	Sonication;
TS	Thermosonication;
OH	Ohmic heating;
CH	conventional heating;
PPO	Polyphenoloxidase;
PME	pectinmethylesterase;
HMF	Hydroxymethyl furfural;
TP	total phenolic contents;
TF	total flavonoids content;
TCC	total carotenoids content;
TPC	total plate count;
PB	Psychrophilic bacteria;
M and Y	mold and yeast.
