# Supplementary material for: Positive Influences of Ohmicsonication on Phytochemical Profile and Storage Stability of Not-from-Concentrate Mango Juice
Source: Molecules. 2022 Mar 18;27(6):1986. doi: 10.3390/molecules27061986 (PMC8950190; doi:10.3390/molecules27061986)
Supplement: Supplementary file 1 [file molecules-27-01986-s001.zip › molecules-1624213 supplementary.pdf]

molecules

SUPPLEMENTARY MATERIAL

Positive Influences of Ohmicsonication on Phytochemical Profile and Storage Stability of Not-From-Concentrate Mango Juice

Tarek Gamal Abedelmaksoud [1,2,*], Sobhy Mohamed Mohsen [1], Lene Duedahl-Olesen [2], Ammar B. Altemimi [3], Mohamed Mohamed Elnikeety [1], Francesco Cacciola [4,*] and Aberham Hailu Feyissa [2]

[1] Food Science Department, Faculty of Agriculture, Cairo University, 12613 Giza, Egypt; sobmohsen1@hotmail.com (S.M.M.); mnoketi@yahoo.com (M.M.E.)
[2] National Food Institute, Technical University of Denmark, 2800 Kgs Lyngby, Denmark; lduo@food.dtu.dk (L.D.-O.); abhfe@food.dtu.dk (A.H.F.)
[3] Food Science Department, College of Agriculture, University of Basrah, 61004 Basrah, Iraq; ammar.ramddan@uobasrah.edu.iq
[4] Department of Biomedical, Dental, Morphological and Functional Imaging Sciences, University of Messina, Via Consolare Valeria, 98125 Messina, Italy
* Correspondence: tareekgamal_88@agr.cu.edu.eg (T.G.A.); cacciolaf@unime.it (F.C.)

Table S1. Ohmic sonication (high power) and PPO and PME activity in mango juice.

Run Order	OH Temperature (°C), χ_1	Sonication Time (min), χ_2	% Inhibition of PPO	% Inhibition of PME
1	75 (+1)	2 (−1)	92.24 ± 1.15	85.14 ± 2.10
2	65 (0)	2 (−1)	77.28 ± 1.71	71.35 ± 1.16
3	65 (0)	8 (+1)	90.14 ± 1.32	83.15 ± 1.27
4	65 (0)	5 (0)	85.59 ± 1.40	72.20 ± 1.14
5	55 (−1)	5 (0)	75.25 ± 1.61	72.54 ± 1.19
6	75 (+1)	5 (0)	98.25 ± 1.45	92.20 ± 2.14
7	65 (0)	5 (0)	86.32 ± 1.16	73.40 ± 1.16
8	55 (−1)	8 (+1)	82.25 ± 1.37	79.51 ± 1.09
9	75 (+1)	8 (+1)	100 ± 1.54	95.49 ± 1.83
10	55 (−1)	2 (−1)	65.25 ± 1.18	58.97 ± 1.15

In the 2nd and 3rd column: the coded values of the test parameters are in parenthesis and the real (un-coded) values are outside the parenthesis; Data are means ± standard deviation (n = 3); PME: pectinmethylesterase; PPO: polyphenoloxidase.

Table S2. ANOVA for Response Surface Quadratic model (PPO and PME of mango juice.)

Source	Coefficient Estimate		Sum of Squares		df		*p*-value	
	PPO	PME	PPO	PME	PPO	PME	PPO	PME
**Model**	85.83	75.02	1032.53	10.68	5	3	< 0.0001	0.0004
χ_1	11.29	10.30	764.78	1.59	1	1	< 0.0001	0.0002
χ_2	6.27	7.12	235.88	8.24	1	1	< 0.0001	0.0015
$\chi_1 \chi_2$	−2.31	-	21.34	-	1	-	0.0003	-
χ_1^2	1.04	-	2.53	-	1	1	0.0140	0.0338
χ_2^2	−2.00	-	9.33	0.85	1	-	0.0013	-
**Residual**	85.83		0.58	0.22	4	6	-	-
**Lack of Fit**	11.29		0.31	0.21	3	5	0.7907	0.1840
**Df = 11**								
**R^2**	0.999	0.943						
**Adj-R^2**	0.998	0.915						
**Pred–R^2**	0.996	0.820						
**C.V. %**	0.45	4.05						
**PRESS**	3.67	193.68						

p-value is significant at $p < 0.05$; $x_1$is coded OH temperature and x_2 is coded sonication time; df: degrees of freedom; CV: coefficient of variation; PRESS: predicted residual sum of squares; PME: pectinmethylesterase; PPO: polyphenoloxidase

In order to select a suitable sonication time and OH temperature intervals, some pre-experiments were conducted between an OH temperatures range of 55–75 °C and sonication time range of 2–8 min for NFC mango juice, with more than 8 min adverse color and ascorbic acid changes identified in orange, apple and mango juices.

On the other hand, increasing the temperature more than 80 °C caused darkness in color, as well as an increase in juice bubbling, which led to loss some of the juice. The voltage gradient of each OS treatment was 40 V/cm according to Abedelmaksoud et al. (2019 a, b). So, the sonication time and OH temperature was selected as 2–8 min and 55–75 °C (mango) for RSM, respectively.

PPO and PME activities are shown in Tables S1 and S2. Optimization of OS parameters was carried out by applying second order polynomial equation (Equations (S1) and (S2)). The regression coefficients for independent variables were obtained by multiple regression analysis.

Tables S1 and S2 show the effect of OH temperature and sonication time on PPO activity and PME activity at 95 % confidence interval using ANOVA analysis. The model shows high significance and good fit with the experimental data of PPO and PME activity, with less variation around the mean [R^2 were 0.999 and 0.943 for PPO and PME, respectively]. The response variability could be explained by the fitted model. The adj-R^2 value (0.998 and 0.915 for PPO and PME) for the model did not differ dramatically compared to R^2 – stating a high degree of correlation between the experimental and predicted values. The lack-of-fit was insignificant ($p > 0.05$). Based on these results, the model was satisfactory for predicting PPO and PME activity under factors within the applied experimental ranges.

The positive linear effect of OH temperature (χ_1) and sonication time (χ_2) were found to be significant for the response variable (PPO and PME) in mango juice, in addition to the interaction (χ_1 χ_2) and the quadratic effect of OH temperature (χ_1^2) on PPO. PME activity were also found to be significant ($p < 0.05$), except for PME (the interaction (χ_1 χ_2) found to be insignificant). However, the quadratic of sonication time (χ_2^2) had an insignificant ($p > 0.05$) effect on the PME, while the quadratic of sonication time (χ_2^2) was significant in PPO.
The insignificant variables were removed and the fitted second order polynomial equation was presented as (Equations (S1) and (S2)):

$$PPO = +85.83 + 11.29\ \chi_1 + 6.27\ \chi_2 - 2.31\ \chi_1\ \chi_2 -.04\ \chi_1^2 + 2\chi_2^2 \quad (S1)$$

$$PME = +75.02 + 10.30\ \chi_1 + 7.12\ \chi_2 + 5.62\ \chi_1^2 \quad (S2)$$

where χ_1: OH temperature (°C) and χ_2: sonication time (min) are the coded values
Second order polynomial models obtained in this study were used for response (PPO and PME activity) to determine the specified optimum conditions. As illustrated in Figure S1, the PPO and PME activity decreased with increasing OH temperature and sonication time. The optimum conditions for OS of NFC mango juice were obtained at the maximum

PPO and PME inactivation by applying the desirability function. The obtained optimum parameters were 75 °C for 8 min in mango juice, (PPO inactivation were 100%), while the PME inactivation were 98.05% in NFC mango juice.

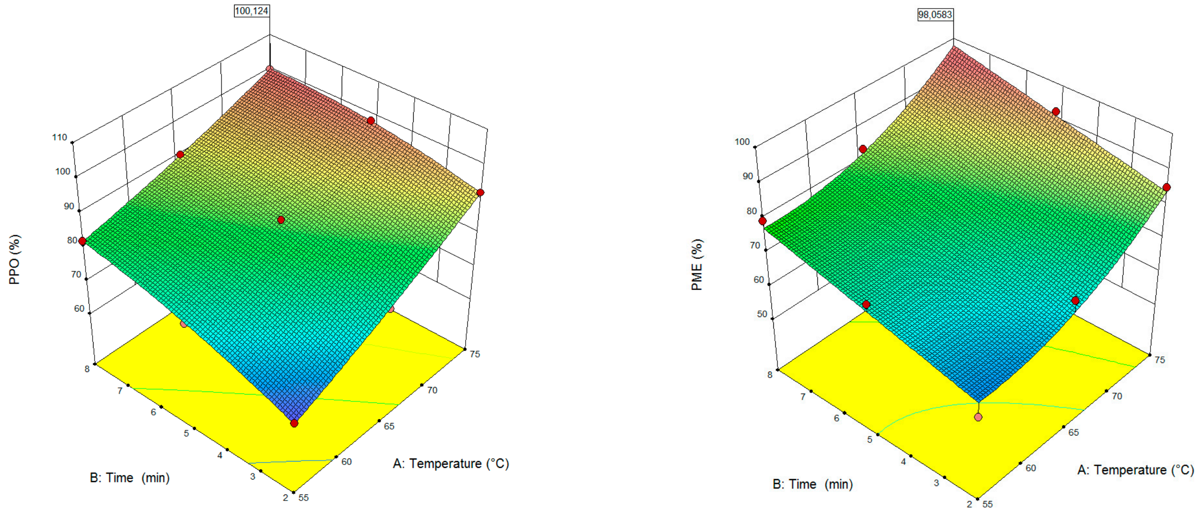

Figure S1. Effect of Ohmic sonication (OS) parameters (temperatures and time) on the PPO and PME activity of mango juice (U/ml/min)—response surface and contour plots. Blue indicates lower PPO and PME activity and red indicates higher PPO and PME activity.
